# Emerging Quantitative Targeted Metabolomics Approaches for Future Inborn Errors of Metabolism Screening

**DOI:** 10.3390/diagnostics16111717

**Published:** 2026-06-03

**Authors:** Shayma A. Osman, Ahmed Malki, Haya Al-Sulaiti, Osama Y. Al-Dirbashi, Mohamed A. Elrayess

**Affiliations:** 1 Biomedical Sciences Department, College of Health Sciences, QU Health, Qatar University, Doha P.O. Box 2713, Qatar; ahmed.malki@qu.edu.qa (A.M.); haya.alsulaiti@qu.edu.qa (H.A.-S.); oaldirbashi@hamad.qa (O.Y.A.-D.); 2 Biomedical Research Center, QU Health, Qatar University, Doha P.O. Box 2713, Qatar; 3 Department of Lab Medicine and Pathology, Hamad Medical Corporation, Doha P.O. Box 3050, Qatar; 4 College of Health and Life Sciences, Hamad Bin Khalifa University, Doha P.O. Box 17666, Qatar; 5 College of Medicine, QU Health, Qatar University, Doha P.O. Box 2713, Qatar

**Keywords:** quantitative targeted metabolomics, inborn errors of metabolism, newborn screening, clinical metabolomics, biomarkers, precision medicine

## Abstract

Inborn errors of metabolism (IEMs) are a group of inherited genetic conditions that, in general, result from a specific enzyme defect. Clinical consequences caused by abnormal enzyme levels often disrupt affected metabolic pathways and their intermediary metabolites. Because diagnostic outcomes depend on early intervention, a timely and accurate diagnosis is essential. Quantitative targeted metabolomics (QTM) is an analytical approach that quantifies predefined metabolites and generates interpretable biochemical phenotypes. Instead of focusing solely on screening, expanded QTM methods enable higher coverage with multi-analyte profiling that can provide more comprehensive characterization of disease-associated metabolic perturbations, particularly in IEMs with overlapping biochemical profiles. This review summarizes diagnostic techniques for IEMs, outlines the principles and advantages of QTM, and evaluates its established role and emerging opportunities and limitations in advancing method development and deep metabolic phenotyping to support precision medicine.

## 1. Introduction

Inborn errors of metabolism (IEMs) are a large group of disorders, commonly inherited as autosomal recessive, that result from defects in metabolic pathways. These abnormalities can lead to either metabolic toxicity or enzyme deficiencies that affect metabolic processes and biochemical products [1]. IEMs are categorized into intoxication disorders, energy metabolism disorders, and storage diseases, in which cellular processing of large molecules is impaired [2,3]. The International Classification of Inherited Metabolic Disorders (ICIMD) classifies more than 1450 disorders into 124 groups and 24 distinct categories [4]. Clinically, IEMs present with a spectrum of symptoms, ranging from mild, such as childhood developmental delays, to aggressive presentations like hyperammonemia, hypoglycemia, and metabolic encephalopathy that require immediate medical attention [2]. Ethnicity, geography, and screening programs are factors that affect the prevalence of IEMs. Nations with cultural practices that favor consanguinity have higher incidence rates of IEMs [5].

To prevent serious and fatal consequences, early diagnosis is crucial. However, clinical heterogeneity and overlapping biochemical profiles among different diseases make diagnosis challenging. In addition, traditional screening tools based on basic metabolite panels may limit accurate disease recognition [6,7]. Many IEMs are treatable, and favorable outcomes are often associated with early detection, underscoring the importance of newborn screening (NBS). NBS helps in preventing irreversible damage and enables the management of asymptomatic newborns before complications arise later in life [8]. Current NBS practices cover the identification of inherited disorders including hematologic, endocrine, and IEMs shortly after birth [9]. Nonetheless, a limited number of IEMs are included in the screening programs, which often depend on each country’s resources and health policies [10]. Consequently, some conditions may remain undetected and present clinically before screening is completed, which can delay appropriate treatments as seen in neonates presenting with seizures [11].

Dietary changes, vitamins, cofactor supplements, pharmaceuticals, enzyme replacements, organ transplantation, and gene therapies are among the available treatments for IEMs [12]. Aligning local data with international standards remains challenging, especially in developing countries. For example, South Asia reports a high number of IEM cases, often linked to high consanguinity rates [13]. However, the absence of comprehensive NBS programs in countries, including Pakistan, results in untreated disorders due to limited funding, expertise, and variable diagnostic capabilities [13]. Advanced analytical technologies such as tandem mass spectrometry (MS/MS) are crucial for detecting treatable IEM disorders by measuring specific biomarkers in biological samples like blood and urine. Implementing these advanced analytical techniques helps establish standardized NBS programs, improving IEM detection and overall health outcomes [2,3]. Traditional MS/MS screening assays for amino acid and acylcarnitines are established forms of quantitative targeted metabolomics (QTM), but they quantify a limited number of preselected metabolites and may not represent the full metabolic complexity of IEMs. To overcome these limitations, QTM has evolved into a comprehensive analytical methodology that provides a thorough evaluation of the metabolome by measuring hundreds of metabolites instead of focusing on a narrow set of screening biomarkers. QTM is a hypothesis-driven measurement of preselected metabolites that uses internal standards, validated methods, and quality controls to provide reliable quantification. Established QTM foundational assays such as amino acids and acylcarnitine have supported newborn screening and IEMs diagnostics [14]. As a confirmatory and investigative approach, metabolomics is well positioned to refine and expand diagnostic methodologies and to inform precision therapeutic strategies based on detailed metabolic profiling [15].

This review article evaluates QTM as a clinical analytical methodology for the screening, diagnosis, and monitoring of IEMs. QTM includes established first-tier FIA-MS/MS amino acid and acylcarnitine newborn screening assays, while expanded QTM refers to the use of broader LC-MS/MS panels and second-tier procedures that cover more metabolite classes with improved specificity through chromatographic separation. We summarize targeted panels ranging from classical amino acid and acylcarnitine to expanded targeted platforms, discuss the advantages and area where expanded coverage may improve IEMs detection, and note practical gaps of QTM that could be addressed for routine clinical implementation.

### 1.1. Metabolomics

Metabolomics employs comprehensive analytical methodologies that are categorized into targeted and untargeted analytical methods with qualitative and quantitative approaches. It enables detailed detection in response to genetic and environmental perturbations [16,17]. Mapping metabolites across diverse classes such as amino acids, fatty acids, and steroids supports the discovery of novel biomarkers and, importantly, optimization of analytical strategies to capture the full spectrum of metabolic alterations in inherited diseases [18,19].

#### 1.1.1. Metabolomics Analysis Workflow

For diagnosis, appropriate biological samples are selected and analyzed depending on the clinical needs, including plasma, serum, urine, saliva, sweat, or cerebrospinal fluid (CSF). In newborn screening, the standard specimen is dried blood spots (DBSs) collected on filter paper to achieve high-throughput analysis. Obtaining these samples, it is necessary to handle them following an optimized sample preparation procedure to avoid degradation of metabolites and analytical variability. This involves steps of metabolite extraction, protein precipitation, and normalization before the initiation of metabolomics analysis [20].

Mass spectroscopy (MS) and nuclear magnetic resonance (NMR) are the main analytical platforms in metabolomics analysis. MS is a commonly used platform in clinical metabolomics for its higher detection sensitivity with broad metabolite coverage. In terms of IEMs, MS is considered more efficient and works in two methods: (1) Flow-injection analysis (FIA-MS), which works by direct injection of the sample into the source of MS ionization; and (2) chromatographic separation, using liquid chromatography (LC-MS) or gas chromatography (GC-MS), allowing for separation of metabolite mixtures by the column before they pass through MS detection for more specific and accurate metabolite detection [21].

Metabolomics techniques are divided into targeted and untargeted approaches. Targeted metabolomics is suitable for clinical diagnostics and newborn screening because it provides quantification of preselected groups of metabolites with high sensitivity. However, untargeted metabolomics can profile a wide range of metabolites in a biological sample without the need for previous knowledge or the selection of specific molecules. This allows for the generation of new hypotheses about disease mechanisms and other biomarker discoveries [22].

Metabolomic studies have a limited number of samples compared with the dataset generated by measuring thousands of metabolites per sample. The application of multivariate statistical analyses and data visualization tools is commonly used to separate metabolic patterns related to diseases and clinical symptoms. Metabolite annotation, pathway analysis, and integration with other omics have been improved by applying bioinformatics and statistical methods, which have increased the translational efficacy of metabolomics in clinical research [23].

#### 1.1.2. Commonly Targeted Metabolites

Quantitative targeted metabolomics in both newborn screening and clinical diagnosis focuses on three major classes of metabolites: amino acids, organic acids, and acyl-carnitines [24]. While amino acids and acylcarnitines are the primary targets in NBS, organic acid analysis is performed in the diagnostic setting through urine profiling. For example, the diagnosis of phenylketonuria (PKU), one of the classical IEMs, is achieved by detecting elevated phenylalanine levels and phenylalanine to tyrosine ratio, which suggest a deficiency in phenylalanine hydroxylase [25]. Enzyme deficiency in amino acid metabolism can lead to the accumulation of upstream substrates in specific pathways. Moreover, urea cycle disorders (UCDs) interfere with nitrogen clearance pathways by converting nitrogen into urea. For example, arginosuccinate lyase (ASL) insufficiency is characterized by elevated citrulline and argininosuccinate with concomitant reduction of arginine levels [26].

Organic acidurias, or organic acid disorders (OADs), are inherited as autosomal recessive conditions. They are characterized by the accumulation of toxic organic acids in the body due to enzyme defects in protein metabolism. Classical examples include iso-valeric acidemia, methylmalonic acidurias, and propionic acidemia. Propionic acidemia, caused by defects in propionyl-CoA carboxylase, leads to the accumulation of methylcitric acid, causing severe symptoms and organ damage [27].

Another class of IEMs is fatty acid oxidation disorders (FAODs), which are identified through NBS by acylcarnitine profiling. Several FAODs are caused by defects in the β-oxidation cycle, which affect cellular energy production. For instance, medium-chain acyl-CoA dehydrogenase (MCAD) deficiency can be identified by the elevation of C8-acylcarnitine and other medium chain acylcarnitines. Clinically, FAODs often present with hypoglycemia in early infancy and can also occur later in life because of metabolic stress [28,29].

## 2. Evolution of Diagnostic Approaches

### 2.1. Traditional Methods

The evolution of newborn screening arose in the 1960s, starting with a simple test for PKU, which detected phenylalanine on dried blood spots (DBSs) [30,31]. Dr. Guthrie, a microbiologist, developed the bacterial inhibition assays for phenylalanine in DBSs with high throughput, allowing the implementation of newborn screening. The field expanded to include other disorders such as congenital hypothyroidism, which relied on calorimetric, radio-immunoassays, and fluorometric assays. These early tests were applied to a single disorder per test [32]. Screening methods to detect IEMs have advanced from a single test for PKU using DBS samples collected on filter paper. Today, these tests have expanded to a comprehensive test that can cover over 50 different metabolic disorders.

Diagnostic metabolomics has evolved through advances in chromatographic techniques. Paper chromatography was one of the earliest methods for metabolic diagnostics, used for amino acids and organic acids identification and separation to detect metabolic disorders. This approach was simple and feasible; however, it had limitations of low sensitivity and poor resolution. Paper chromatography was used mainly to analyze amino acids in urine for broad screening, and GC-MS was used for high precision to quantify amino acids present in serum [33]. GC-MS became the gold standard for the analysis of urinary organic acid in IEMs confirmatory tests such as organic acidurias [34]. With the need for more sensitive and reproducible platforms, high-performance liquid chromatography (HPLC) was one of the confirmatory tools before the widespread use of current MS/MS platforms that offer precise quantification of amino acids, purines, and neurotransmitters in plasma, urine, and CSF [35]. These developments of chromatography techniques have also transformed NBS platforms. The main shift was made possible by the triple quadrupole MS/MS, which changed the technology into a standard clinical setting [36]. In the late 1990s, a significant transformation occurred with the introduction of electrospray ionization MS/MS, which enabled the measurement of metabolites from a single sample (multiplex metabolite techniques) [37]. Therefore, the paradigm of one-spot, one-test, one-disorder has changed into one-spot, one-test, many-metabolites for different disorders, which led to the development of screening panels that cover from 10 to 25 disorders [30]. In 2006, the Recommended Uniform Screening Panel (RUSP) was defined and standardized by the American College of Medical Genetics (ACMG) to have a uniform NBS panel. This panel identified 29 core conditions and other 25 secondary targets that support differential diagnosis of the core conditions [32]. By 2010, the United States Secretary of Health and Human Services officially established the RUSP using an ACMG report as the foundation for standardized national NBS [38]. Current NBS programs rely on MS/MS as the primary analytical platform for NBS [31] because it provides high-throughput and multiplexed detection that are needed for efficient population screening. GC-MS is used in confirmatory diagnostic testing of metabolic disorders such as organic acidurias and has lower throughput compared with MS/MS [39,40].

Modern NBS programs have been enhanced to improve screening and reduce false-positive results [30]. Additionally, by separating chemicals before ionization, reducing isobaric interferences at constant pressure, and enabling the quantification of specific metabolites, LC-MS/MS has improved the accuracy of NBS [41]. For example, according to Mak et al. (2023), the LC-MS/MS panel is used as a second-tier test (follow-up analysis) that reduces false-positive results across different disorders while maintaining test sensitivity [39]. Traditional NBS testing has evolved by the incorporation of next-generation sequencing (NGS), or whole-exome sequencing (WES), with biochemical methods. Recent studies have indicated that combining MS/MS screening with sequencing techniques has reduced false positives by providing genetic findings to be checked across a panel of 121 metabolites [42]. Also, in the identification of pathogenic variations in a group of 2350 genes, WES-based methods added value to the confirmatory diagnosis following abnormal NBS results of IEMs compared with traditional panels. Nevertheless, challenges of ethics, finance, and data interpretation still exist [43]. Table 1 provides a concise timeline of the technological shifts from 1960s bacterial inhibition assays to the 1990s MS/MS revolution that transformed into high-throughput and multiplexed technologies.

### 2.2. Metabolomics Challenges

The complexity of metabolic pathways and differences in testing technologies can reduce the overall efficacy and efficiency of NBS programs. The high rate of false-positive and false-negative results is one of the main challenges recorded in metabolomics studies. False-positive results can lead to parental anxiety, unnecessary follow-up testing, and even overtreatment of mild conditions [54]. On the other hand, false-negative results are more harmful, because affected infants will miss a timely diagnosis, delaying critical medical interventions. The biochemical profiles of overlapping IEMs and the dynamic metabolite levels of newborns are the main causes of these problems.

In diagnostic settings, identifying ultra-rare or novel disorders remains challenging. While NBS panels are designed to screen for conditions that have known biomarkers and available treatments, the diagnostic setting can fail to identify patients with ultra-rare or novel IEMs. Those conditions are poorly characterized or not yet represented in existing diagnostic panels. As a result, patients who are suspected of having IEMs may go undiagnosed, leading to severe clinical outcomes later in life. In NBS programs, the interpretation of variants of uncertain significance (VUSs), cost, and infrastructure are the challenges that limit the combination of NGS and genomics into screening programs [55]. Logistic and operational delays also weaken NBS effectiveness. Insufficient sample collection, false-negative results, or diseases that manifest later in life all can lead to delayed diagnosis in NBS. Timely diagnosis management in NBS can also be obstructed by practical challenges such as delayed sample delivery, lack of trained healthcare professionals, and ineffective tracking and communication systems for isolated regions [56].

## 3. Quantitative Targeted Metabolomics (QTM): Principles and Advantages

Beyond the application of QTM in NBS panels, it has evolved into a platform for improving analytical performance and systematically characterizing disease-related metabolic networks in IEMs. QTM combines analytical accuracy with mechanistic interpretability of metabolic perturbations [57]. In current routine NBS, QTM is frequently implemented by FIA-MS without chromatographic separation to achieve high-throughput analysis of amino acids and acylcarnitines in a single run [58]. Beyond screening, method development efforts increasingly focus on adapting and extending these QTM workflows, for example by incorporating chromatographic separation or broader metabolite panels, to obtain a more comprehensive view of disease-related metabolic changes [58].

### 3.1. Core Technology: LC-MS/MS for Defined Quantification of Metabolites

LC-MS/MS is the central platform in quantitative targeted metabolomics that supports the detection of IEMs with high resolution and more sensitive quantification of metabolites obtained from DBSs or urine. It is commonly combined with triple-quadrupole instruments using multiple reaction monitoring (MRM) or selected reaction monitoring mode (SRM). This combination enhances chromatographic separation and analyte ionization. This allows selective and sensitive detection of both polar and nonpolar metabolites at various concentrations within one analytical run [59].

Quantification in LC-MS/MS techniques depends on calibration curves and the use of stable isotope-labeled internal standards (SIL-ISs). These internal standards compensate for the matrix effects that occur during sample preparation or coeluting chemicals that cause variability in the analysis of metabolites [60]. The coefficient of variation (CV) is one of the ways used to express the precision of QTM measurements. Matching SIL-ISs of each analyte produced a consistent median resulting in low between-run CVs (2.7–5.9%) in a cross-platform validation for more than 70 biomarkers, even with variations in analyte retention times [61]. Clinically, low CVs are essential to maintain high analytical precision and improve diagnostic accuracy by reducing false-positive or -negative outcomes.

Combining hydrophilic interaction chromatography (HILIC) with MS/MS supports a broad second-tier screening method to be used directly from DBSs. This multi-tier approach can increase assay specificity and expand metabolite coverage by the detection of multiple biomarker classes such as organic acids and lysophosphatidylcholines [62]. In addition, using two SRM transitions per analyte may improve accurate identification. Recent LC-MS/MS techniques have identified 235 metabolites from 17 chemical classes [63]. The use of such advancements demonstrates that QTM is a reliable analytical technique not only for clinical interpretation within NBS platforms but also for refining laboratory methodologies and expanding metabolite coverage to capture more complete metabolic fingerprints of IEMs.

### 3.2. Key Features of QTM and Their Diagnostic Impact

#### 3.2.1. Expanded Multi-Analyte Coverage

QTM can measure hundreds of metabolites across different classes, including amino acids and diverse lipid species, beyond traditional targeted panels [57]. This expanded multi-analyte coverage enables the development of more informative assays that capture broader pathway disturbances in IEMs in a single workflow, rather than being limited to a narrow set of screening markers. For example, a validated LC-MS/MS assay can quantify 721 metabolites using calibration curves and SIL-ISs to confirm analytical accuracy and reproducibility [57]. Modern platforms have reported panels that went beyond 2000 metabolites, showing a quick development of large-scale metabolite profiling. The validation of each metabolite detection remains a critical requirement for clinical applications with respect to limits of detection [64]. The use of QTM in IEMs diagnosis can target specific metabolites that are not covered by the core panels used in first-tier newborn screening. Table 2 connects QTM metabolite targets and shows how larger coverage enhances specificity in confirming IEM groups. It summarizes how targeted metabolic classes can help reduce false positives by supporting second-tier techniques and improving specificity in diseases with overlapping biochemical profiles. However, accurate quantitative validation and the interpretation of data within standards are essential in determining the therapeutic value of extended target lists.

#### 3.2.2. Analytical Precision for Reliable Diagnosis

The detection of small biochemical changes in biological specimens needs techniques with high analytical precision. The patient’s outcomes are very sensitive to minor analytical errors that can affect case classification and lead to false results of the disease. CVs are used to validate QTM assays, and values lower than 10% are considered acceptable and valid for NBS applications. This threshold confirms that analytical variability is low enough to distinguish between healthy and affected newborns. Regulations are strict about CVs thresholds, especially the ones close to clinical cutoffs that reflect an actual biological change rather than technical noise. In addition to precision, validation of QTM assays needs examination of accuracy and trueness to verify that it is aligned to the reference data and to lower systematic bias [67]. A large quality assurance study involving 302 newborn screening laboratories have tested the same metabolites using constant quality control materials. They found that the results are more consistent and complemented across different laboratories, which reduces inter- and intra-laboratory variability and sets out a reliable cutoff decision for metabolites such as phenylalanine. The consistency of results matters because NBS decisions are critical and cannot be ambiguous. Therefore, the higher the analytical imprecision is, the higher the risk of false results [68]. The precise measurement of metabolites depends on both the analyte’s chemical characteristics and how the assay is designed. For instance, the quantification of 17α-hydroxyprogesterone (17α-OHP) is a marker for congenital adrenal hyperplasia. The isotope-dilution LC-MS/MS candidate reference measurement procedure (cRMP) measurement provides low CVs of 1.27–5.69%. However, other immunoassays usually have higher false-positive results. Controlling matrix effects and determining limits of detection/quantification (LOD/LOQ), recovery, and linearity can also provide accurate measurements of steroid hormones using isotopes based on calibration [65].

#### 3.2.3. Data Normalization and z-Score Interpretation

Normalization using internal standards and reference populations improves the interpretability of metabolites through metrics such as z-score. The Metchalizer method, for example, forces internal standards to normalize metabolite variations, allowing calculation of age- and sex-adjusted z-scores. Regression-based covariates can enhance detection of abnormal biomarkers. By minimizing technical variability, QTM ensures that measured metabolites represent true and actual biological differences, thereby enhancing accuracy and reliability of diagnostic interpretation [69].

#### 3.2.4. Reference Intervals for Clinical Context

Reference intervals (RIs) are one of the clinical laboratory tools for data interpretation. It is applied to check if the lab results are within or outside the reference interval. Metabolic analysis of newborns can vary with the metabolite levels changing after birth. Therefore, NBS labs are establishing RIs that work as a population baseline for z-score grouped by age, sex, gestational age, and birth weight. For example, a study has confirmed that RIs of 35 MS/MS NBS biomarkers that have been generated with stratified postnatal age were able to enhance specificity during the first week of life [70]. Additional studies have also defined RIs for amino acids and acylcarnitine in DBS samples, contributing to helping clinicians with precise classification of metabolic disorders.

#### 3.2.5. Managing Preanalytical Variability

In DBS-based QTM workflows, analytical variability and imprecision can arise from preanalytical and matrix factors such as hematocrit variability, humidity, and temperature during transport and storage [71]. Hematocrit affects blood spot viscosity and analyte distribution on DBS cards. Increased levels of hematocrit lead to high viscosity, which provides uneven spreading that can result in less uniform analyte extraction and smaller spot diameter [72]. Recent studies have demonstrated that a 1% increase can lower the concentration of some analytes, such as thyrotropin (TSH) measured in the eluate, resulting in false-positive/-negative results if the analysis is not corrected [73].

Changes in baseline metabolite levels can result from variations in sample collection times relative to birth (e.g., for neonates; the recommended timing is 24 to 48 h) [32]. To handle this, laboratories with QTM implemented are advised to include standards and quality control (QC) measures. Also, including the participation of external QC, multi-concentration QC material, and internal standards can maintain confidence in the test performance [74]. Although standardized QTM can reduce preanalytical variabilities, additional factors are discussed in the challenges and limitations section.

### 3.3. Targeted vs Untargeted Metabolomics: Complementary Approaches

Quantitative analysis, high sensitivity, direct clinical interpretation, and standardized repeatable methods are some of the quantitative targeted metabolomics (QTM) benefits. Targeted and untargeted metabolomics have different but complementary uses rather than competing methods. In NBS, targeted methods are required and mandatory to screen for conditions with known biomarkers and available treatments. In diagnostics and research settings, the use of untargeted methods is more valuable for hypothesis generation and novel biomarker discovery.

#### 3.3.1. Absolute Quantification and Sensitivity

Small biological changes can be reliably detected by QTM, which provides precise measurements of chemically defined metabolites. Targeted LCMS/MS panels, for example, can measure low-abundance metabolites in DBSs, such as amino acids, which are frequently missed by untargeted workflows [75]. The accurate classification of affected patients and unaffected ones is improved by this high sensitivity of QTM.

#### 3.3.2. Clinical Interpretability

Targeted metabolomics focuses on metabolites with proven clinical relevance; its results can be immediately used to make diagnostic decisions. Untargeted metabolomics supports the identification of new biomarkers and allows hypothesis-driven discovery for novel markers, complementing targeted approaches in research [76]. For example, targeted assays have been used to profile COVID-19 patient serum, yielding useful biomarker data suitable for therapeutic and prognostic stratification [20].

#### 3.3.3. Standardization and Reproducibility

The processes of QTM are highly standardized, with strict quality control, calibration, and validation measures. By reducing technical variability, these procedures guarantee consistent results across samples, instruments, and laboratories. While untargeted techniques offer broader metabolite coverage, they are more appropriate in research since their broader coverage overcomes the need for strict analytical standardization [75,77].

#### 3.3.4. High Throughput and Efficiency

Targeted panels focus on a predefined set of metabolites, providing compact datasets that are easier to interpret and process. This allows rapid turnaround time, making QTM applicable for screening programs. Triple acquisition mass spectrometry (TRAM) and simultaneous quantitation and discovery (SQUAD) are some of the developing hybrid techniques that broaden the metabolic coverage. Moreover, they can maintain quantification with high quality, which makes them primarily appropriate for research and discovery settings rather than routine clinical diagnostics [78]. These advantages together show the ability to provide precise quantification using standardized techniques established for diagnostic applications. The development of hybrid techniques such as TRAM and SQUAD can broaden metabolic coverage and discovery, while targeted panels can provide rapid and consistent quantification. Table 3 summarizes the key differences between targeted and hybrid approaches, where targeted metabolomics is the standard for clinical tests and hybrid techniques offer broad coverage.

## 4. Clinical Applications of QTM in IEM Diagnosis

### 4.1. Newborn Screening

Limitations of traditional screening platforms are increasingly addressed by QTM-based workflows that provide more extensive coverage. From a single DBS sample, hundreds of clinically relevant metabolites can be quantified using tandem mass spectrometry, which enables both high-throughput analysis and more comprehensive profiling of disease-related metabolic pathways. In addition to traditional amino acid and acylcarnitine panels, such expanded metabolite sets support improved characterization of lysosomal storage diseases, peroxisomal disorders, and other IEMs [46]. A multi-analyte validation study measuring more than 200 plasma metabolites showed reproducible performance platforms suitable for metabolic screening, with intra-assay CVs of 5.7–6.9% and inter-assay CVs up to 12.6% [66].

These analytical advancements are applied in clinical practices and large population studies, which can reflect regional differences in IEM incidence driven by cultural practices and local genetic profiles. For example, a nationwide MS/MS-based NBS program in Qatar (2010–2023) assessed 351,223 neonates and found 318 cases of biochemically confirmed IEMs, resulting in a general incidence of 1 in 1105 births. Amino acid abnormalities were the most common category discovered, followed by organic acidurias, urea cycle disorders, and fatty acid oxidation defects. Due to a single well-characterized founder mutation in the population, classical homocystinuria showed the highest incidence detected among identified disorders, further impacted by cultural endogamy and the favor of consanguinity reported in more than 60% of affected cases [79]. In contrast, a comparable study from Shanghai, China, a 19-year MS/MS-based NBS program, screened about 1.17 million newborns, with 392 confirmed IEM conditions. These included a total of 28 different IEMs, of which around 50% had amino acid disorders, 30% had organic acid disorders, and 20% had fatty acid oxidation defects [80]. This difference in IEM distribution reflects diverse genetic profiles in Chinese population compared with the localized founder effect seen in Qatar, emphasizing the need for regional and specific geographic screening interpretations for effective prevention strategies.

### 4.2. Diagnosis of Complex Cases

Beyond NBS, QTM is a useful diagnostic technique in complex IEM cases, where it can complement genetic screening to provide definitive diagnosis for overlapping or nonspecific clinical symptoms. In mitochondrial disorders, metabolomic profiling has revealed alterations in energy metabolism, amino acid turnover, and sphingolipid molecules. For example, untargeted metabolomics revealed a sphingolipid signature that helped to clarify the diagnosis of a patient with mitochondrial disease (RARS2) who had unusual neurological symptoms [81]. Similarly, neurotransmitter metabolite dysfunction can be detected using cerebrospinal fluid metabolomics. In patients with encephalopathies and other neurological disorders, the use of metabolomics has improved the identification of biochemical phenotypes in disorders that affect neurotransmission [82,83].

### 4.3. Monitoring and Therapy Optimization

In addition to diagnosis, metabolomics supports long-term metabolic monitoring and assessment of IEM patients through early detection of metabolic instability to guide treatment decisions [16]. Patients treated for glucose transport 1 deficiency syndrome (GLUT1-DS) have been monitored with the use of untargeted metabolomics. A study used metabolomic profiling to measure the metabolic change before and after starting a ketogenic diet, using plasma, urine, and CSF. Metabolomics can monitor therapeutic effects and investigate neurometabolic disorders, as demonstrated by the results, which revealed that metabolite shifts were correlated with the treatment [17].

Furthermore, next-generation metabolic screening (NGMS) integrates untargeted metabolomics with standardized computational pipelines. Clinicians can use these platforms to identify secondary metabolic disorders, monitor treatment responses, and modify personalized diet or medication regimens [84,85].

## 5. Integration and Multi-Omics Approaches

### 5.1. Genomics Correlations

**Figure 1:**
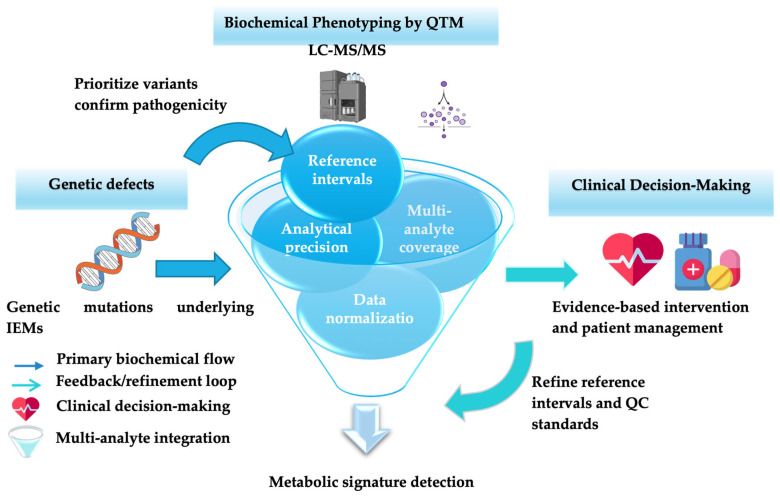
Continuum of precision diagnostics that integrates genomics, QTM biochemical phenotyping, and clinical decision-making.

QTM can be integrated with genomic sequencing such as whole-genome sequencing (WGS) or WES to provide functional biochemical context for variant interpretation in IEM patients. This combined approach helps clinicians determine if variants of uncertain significance result in quantifiable metabolic dysregulation, strengthening variant classification. According to Graham et al., the combination of metabolomics and genomics enhances gene prioritization by showing compatible metabolic patterns in rare or unusual metabolic diseases [86]. Applying this method reveals metabolite abnormalities that match the predicted pathway dysfunction. Moreover, a cohort of 170 patients participated in untargeted metabolomic profiling and exome sequencing. This integration has moved beyond the testing stage, with real-world application showing a 12% increase in diagnostic yield, which helped to interpret variants in 43.5% cases [87]. The functional integration of multi-omics is shown in the diagram of precision diagnosis in Figure 1. The model demonstrates precision diagnostic flow where the biochemical phenotyping by QTM uses LC-MS/MS, which complements genomic findings to support variant classification, confirmatory testing, and clinical decision-making. This framework is applicable as a follow-up or confirmatory technique rather than being used as the first screening test for diseases.

### 5.2. Proteomics and Flux Analysis

Understanding the mechanistic elements of pathway dynamics in IEMs requires the integration of QTM with proteomics and metabolic flux analysis. Proteomics have a role in connecting if these metabolic changes are related to enzymatic levels of variations or post-translational modifications. Fluxomic uses stable isotope tracers to monitor metabolite movement, detecting any enzymatic blockage or compensatory rerouting in enzyme deficiencies while revealing pathway activity and energy metabolism changes [88]. According to Driesen et al., fluxomics provides information about how metabolic pathways operate in diseases. By combining metabolomic, proteomic, and fluxomic data, clinicians can correlate gene defects to downstream metabolite accumulation and enzyme dysfunction. Multi-omics integration is beneficial in complicated IEMs, such as lysosomal storage disease, mitochondrial disorders, and amino acidopathies, where a single omics technique is not enough for diagnosis or interpretation [17].

## 6. Challenges and Limitations

### 6.1. Preanalytical Variability

The significant preanalytical variability that occurs during sample collection, processing, and storage is a major limitation of QTM. Sample collection steps should be handled carefully to prevent metabolite degradation, oxidation, or changes in enzymatic activity. Moreover, if samples are not analyzed quickly, stored at right temperatures, or exposed to freezing and thawing multiple times, metabolite concentrations can change and result in false diagnostic patterns. The variation in sample pH and enzymatic composition between blood, urine, and DBSs can affect how metabolites are extracted and detected by electrospray ionization. Therefore, matrix-specific standardization is essential for reliable and accurate quantification across different metabolite classes [89].

Metabolite levels are changing normally due to physiological and environmental factors such as fasting, circadian rhythm, dietary, and gut microbiota. Disease-specific patterns frequently overlap with these variations in metabolite levels. This makes it challenging to interpret metabolite abnormalities seen in complex diseases such as IEMs [88]. To minimize this risk and guarantee reproducible diagnostic validity, strict preanalytical standardization is necessary, including consistent timing of sampling and anticoagulants use [1].

### 6.2. Data Interpretation and Analytical Constraints

Metabolite levels are impacted by biological, environmental, and technical factors; as a result, data interpretation in metabolomics continues to be a major challenge. In many untargeted metabolomics studies, a large number of detected spectral features are unannotated or not classified as known metabolites. Despite the improvements in databases and annotation tools, there are still significant gaps in the resolution and interpretation of these metabolites and assigning them to defined metabolic pathways. As a result, applying metabolomic data in clinical bases remains a limitation in the field [90].

Furthermore, data interpretation is complicated by analytical limitations in bioinformatics workflows and metabolite identification methods. Variations in metabolite annotation methods, database coverage, and spectral matching strategies can lead to inconsistent metabolite alignment that complicates biological interpretation [91]. Moreover, metabolomics lacks a single unifying approach for metabolite identification, and it needs multiple MS-based data for accurate annotation, including patterns of MS/MS data, chromatographic retention times, and collision cross-sections to match metabolite identities [91].

A major challenge of targeted metabolomics panels is the incapacity to detect disorders that fall outside the predefined scope. Targeted panels measure only known and preselected metabolites; therefore, any novel biomarker will remain undefined within established pathways, resulting in false-negative outcomes [21]. This limitation is relevant as the identified IEMs expand, and other new disorders may not yet be represented in current existing panels [92]. Targeted metabolomics cannot produce hypothesis-driven findings, making it important to combine targeted metabolomics with untargeted metabolomics techniques in order to resolve undiagnosed cases [93].

### 6.3. Cost, Availability, and Implementation Barriers

Financial barriers and infrastructure are the main difficulties that limit metabolomics implementation in routine clinical practice. Advanced computational resources, specialized laboratory infrastructure, and major investments in high-resolution MS platforms are necessary for the application of clinical metabolomics. Consequently, metabolomics services are currently limited to specialized academic or reference laboratories, rather than being applied in routine hospital diagnostic settings [94].

Additional limitations are the absence of standard operating procedures, certified QC materials, and data analysis pipelines that align with clinical laboratory accreditation requirements. Differences in metabolomics platforms, inconsistent data analysis techniques, and the semi-quantitative results of untargeted metabolomics lower the reproducibility of data between laboratories. The transfer of metabolomics into clinical testing is limited by these factors, which makes QC and analytical validation more challenging and delays the alignment to regulatory approval rules [95]. Metabolomics is more costly than traditional biochemical assays due to infrastructure requirements and high-resolution instrumentation needs for clinical use. Despite higher costs, early detection through MS/MS screening improves health outcomes by reducing long-term mortality and morbidity rates, demonstrating favorable cost-effectiveness [31].

## 7. Future Directions

### 7.1. Automation and AI

The large and complex datasets produced by metabolomics require advanced analytical tools, making artificial intelligence (AI) and machine learning (ML) the primary approaches for future clinical translation. According to recent research, supervised learning techniques like neural networks and support vector machines are used for biomarker discovery and disease classification in high-dimensional datasets [96,97]. The interpretable ML models applied to metabolomics data can achieve accurate disease prediction while identifying metabolite signatures. Similarly, explainable automated ML techniques can identify disrupted metabolic pathways that are directly linked to the pathophysiology of diseases while integrating metabolomics data for reliable and accurate diagnosis [98].

### 7.2. Personalized Panels

One important future direction is the design of personalized metabolomic panels tailored to individual genetic backgrounds for precision management of IEMs. Advances in precision medicine emphasize not only integration of genomic data with downstream functional omics, such as metabolomics, but also systematic refinement of analytical panels to capture the full spectrum of patient specific metabolic changes, thereby improving diagnostic resolution and treatment stratification [99].

Untargeted and semi-targeted metabolomics can detect broader biochemical disturbances than traditional single-analyte screening, enabling the detection of novel metabolic phenotypes. Personalized genome-scale metabolic modeling methods for IEMs were reported by Heinken et al., who showed how individual genetic abnormalities can be applied into patient-specific changes in metabolic fluxes [100]. Moreover, the integration of metabolomic profiles with genomic data improves variant interpretation, supports reclassification of variants of uncertain significance, and allows more individualized clinical diagnosis [101]. These findings enhance the development of personalized metabolomics panels, which are interpreted along with genomics to enable more precise and patient-specific diagnosis, monitoring, and management of IEMs.

### 7.3. Point-of-Care Platforms

To make metabolomics more available and accessible outside centralized facilities, point-of-care (POC) and smaller, portable MS platforms are developed. These systems currently offer adequate analytical performance for clinical use, despite the cost of traditional MS platforms. POC metabolic analysis can be a small ion trap-based MS system that can analyze up to 100 metabolites from clinical samples as a real-time profiling [102]. In addition, recent research shows that miniature MS technology is advancing rapidly, and they could be used as a clinical POC applied for quick biomarker detection and therapeutic monitoring outside traditional laboratory settings [103]. MasSpec Pen is another emerging POC platform that offers metabolite monitoring at the bedside. In less than 10 s, a 3D-printed sampling tip is connected to LC-MS/MS for non-destructive molecular extraction. This platform can provide real-time flux measurements necessary for intensive care or patients with complex IEMs. However, challenges including poor spectral resolution and lack of standardized data must be addressed before clinical implementation [104].

### 7.4. Harmonization, Standardization, and Global Access

Harmonization of protocols across laboratories is important for the transition of metabolomics into clinical practice as this will avoid variation in lab results, and without it, findings cannot be meaningfully compared. Preanalytical processes such as specimen collection, storage conditions, and other factors including processing methods can limit the comparability between labs and reduce the feasibility of multi-center studies [105]. To overcome these limitations, establishing standard operating procedures that are internationally agreed would improve reproducibility and support large-scale cooperative IEM research [106]. The harmonization of the data reported and having a standard reporting format is essential to have relevant comparisons between institutions and support the establishment of population representative reference databases [107]. The ERNDIM external quality assurance program aims to build standardized procedures for diagnosis in metabolic laboratories; however, the extension and broader applications in metabolomics remain limited [108]. In addition, equal global access to advanced metabolomic platforms is needed, but limitations of financial constraints and technical complexity can prevent worldwide implementation of metabolomics into routine clinical practice [109].

## 8. Conclusions

QTM provides direct biochemical evidence that connects genetics to clinical manifestations and facilitates a more comprehensive understanding of metabolic derangements in IEMs. QTM can complement other omics such as genomic testing by providing functional biochemical data, enabling more robust methodological frameworks for classifying patients with complex metabolic disorders. The implementation of QTM can support precision therapies based on patient’s metabolic phenotypes. Finally, consistent QTM biomarker panels and methodologically robust workflows that meet regulatory standards support clinical translation of metabolomics toward routine diagnostic use within precision medicine.

## Figures and Tables

**Table 1:** Timeline of MS/MS and emerging metabolomics technologies in newborn screening and IEMs.

Approximate Start	Technique Used	Status for NBS and IEMs	Notes	Citations
1960s–1980	Guthrie bacterial inhibition assay for PKU and a few single-analyte tests	Established (historic core)	PKU became the gold standard model for screening	[44]
1990s	Tandem mass spectrometry (MS/MS)	Established current standard	Applied on dried blood spots (DBSs) for amino acids and acylcarnitine. Low-cost, highly sensitive, and specific in the identification of more than thirty IEMs for many of conditions	[45,46]
Early 2000s–2010s	Expanded MS/MS panels	Established and still expanding	Use of analyte ratios with good clinical outcomes and cost-effectiveness for national and regional programs	[47,48,49]
2000s–2010s	Routine second-tier biochemical tests (the use of LC-MS/MS for homocysteine, urine profiling)	Established adjunct	Used to reduce false positives and improve positive predictive value	[50]
2010s	Targeted next-generation sequencing (NGS)/gene panels as confirmatory tests	Established diagnostic tool	Standard for confirming many IEMs and for genotype–phenotype correlation, not primary screening	[51]
Late 2010s–2020s	NGS as second-tier screening for selected IEMs	Emerging	Marked reduction in false positives and can rescue some false-negative biochemical results	[52]
2020s	Whole-exome sequencing (WES) as alternative primary screen	Not yet proven	WES sensitivity 88% and specificity 98.4%, inferior to MS/MS (99.0% and 99.8%) better suited as secondary test	[53]

**Table 2:** Expanded QTM targets (beyond core panels) and their diagnostic value in IEMs workflows.

Added Metabolite Class	Example Biomarkers	IEM Groups Improved (Examples)	Why First-Tier May Miss/Misclassify	Best Clinical Role	References
Organic acids and lysophosphatidylcholines (LPCs) in a single second tier	Methylcitric acid; selected urinary/DBS OAs; LPC species	Organic acidurias: lysosomal/peroxisomal conditions (not covered in core panels)	First-tier FIA-MS/MS lacks separation; limited marker set with overlapping profiles that can increase false positives	Second-tier NBS/confirmatory	[46,62]
Steroid hormones (quantitative LC-MS/MS)	17α-hydroxyprogesterone (17α-OHP)	Congenital adrenal hyperplasia (CAH) follow-up	Immunoassays have higher false positives; LC-MS/MS improves specificity/precision	Second-tier confirmatory	[65]
Multi-class panels (amino acids, lipids, and others)	Amino acids, acylcarnitines, sphingolipids	Complex/overlapping phenotypes	Narrow first-tier biomarkers may not resolve overlapped profiles or capture disease signatures that extend beyond amino acids and acylcarnitines	Confirmatory/phenotyping	[63,66]
High coverage targeted panels	Large, targeted panels	Broad hypothesis-driven phenotyping; coverage of multiple pathway disturbances	Expanded metabolite coverage alone does not yield in diagnosis. It should be linked to IEM-specific signatures; the clinical barrier becomes validation (LOD/LOQ, linearity, QC, reference intervals)	Second-tier/confirmatory panels	[57,64,67]

**Table 3:** Comparison of targeted and hybrid metabolomics approaches.

Feature	Targeted Metabolomics	Hybrid Metabolomics
Goal	Precise quantification of predefined metabolites	Quantify predefined metabolites while also detecting additional/unknown features
Coverage	Limited but clinically focused panel	Broader coverage (targeted and untargeted features)
Accuracy/Precision	Very high (validated quantitative methods)	High for targeted analytes; semi-quantitative for discovery features
Workflow complexity	Low	Moderate to high (added acquisition and downstream processing)
High-throughput suitability	Excellent (adjusted for quick clinical reporting)	Lower to moderate (more complexed processing/interpretation)
Clinical readiness	Proven for clinical diagnostics and newborn screening	Mostly for research, requires more validation for routine diagnostics
Examples	Amino acid/acylcarnitine panels; PKU screening	Targeted–untargeted hybrid methods (example: TRAM, SQUAD); data-dependent MS combined with targeted quantification

Abbreviations: MRM, multiple reaction monitoring; SRM, selected reaction monitoring; PKU, phenylketonuria; TRAM, targeted ratio analysis metabolomics; SQUAD, simultaneous quantitation, and discovery.

## Data Availability

No new data were created or analyzed in this study. Data sharing is not applicable to this article.

## Abbreviations

The following abbreviations are used in this manuscript:

CV	Coefficient of variation
DBSs	Dried blood spots
IEMs	Inborn errors of metabolism
LC-MS	Liquid chromatography mass spectrometry
ML	Machine learning
MS	Mass spectrometry
MS/MS	tandem mass spectrometry
NBS	Newborn screening
NGS	Next generation sequencing
PKU	Phenylketonuria
POC	Point-of-care
QC	Quality control
QTM	Quantitative targeted metabolomics
WES	Whole-exome sequencing
